# Medicine

**Published:** 1898-09

**Authors:** R. Shingleton Smith


### Progress of the Medical Sciences.

### MEDICINE.

No apology is needed for recurring to the subject of **tuberculosis**, inasmuch as it is of perennial and increasing interest, and the June number of the *Practitioner* was devoted entirely to it. The editor thereof urges a new crusade against tuberculosis, and supplies some matter for the eloquence of a preacher of such crusade.

The Weber-Parkes prize essay, "Researches on Tuberculosis," by Dr. A. Ransome gives charts showing the steady fall in the death-rate from phthisis in England and Wales during the period 1838–1894, and states that "if phthisis diminishes at the same rate during another thirty years it will have entirely disappeared by the end of that period." Taking all forms of tuberculosis together, the decrease means the saving of at least 75,000 lives every year, or three-quarters of a million within the last decade.

It has been frequently pointed out before, and Dr. Ransome emphasises the fact, "that from the results of *post-mortem* examination it appears that from 20 to 30 per cent. of all persons dying in hospitals, between the ages of 25 and 75, show signs of healed tuberculous lesions." Again, it is the usual experience that "most men in fairly extensive practice could point to several persons who have been in the grip of phthisis, and who have been enabled to shake it off." Numerous are the illustrations of this, and it is a matter of daily observation at phthisical sanatoria that the directors and physicians are usually men who have good reason for the faith which is in them by having themselves been cured.

Dr. A. Ransome[1] discusses the **susceptibility** to tuberculosis under different conditions. He points out the fact that no race of mankind can be said to claim exemption; that under certain conditions of environment individuals of every race may contract the disease; that, "given a sufficient dose of actively virulent tubercle bacilli, and the possibility of contracting inflammatory affections of the lungs, no man can be considered

1 *Practitioner*, 1898, lx. 574.

safe from infection by this organism." It is also a disease of all countries and of all climates, extending over every part of the habitable globe: there is obviously nothing surprising in our thus finding the disease wherever human beings are gathered together, as the disease might be expected to follow mankind in its distribution over the surface of the globe. He considers it highly probable that heredity has much less to do with consumption than is commonly supposed, and that none of these influences, with the exception of the soil, have much to do with its spread: it has, however, been abundantly shown that dampness in the subsoil of dwellings does foster the disease, and that good drainage diminishes its prevalence.

The bacteriology of tuberculosis is described by Dr. G. Sims Woodhead.[1] Dr. Allan Macfadyen[2] points out the need for **early diagnosis** of this infectious disease, whether in man or in animals, if we are to combat its stealthy spread, and to limit the dangers that exist from the two great channels of infection, the sputum of phthisical patients and the milk of diseased animals; he states that "the use of tuberculin as a diagnostic agent is of the greatest value, and the neglect of its aid is inexcusable." Good results in diagnosis are being obtained not only from the tuberculin test but also from the Röntgen rays. As regards the former, Dr. Loomis states that tuberculosis can be diagnosed in the human subject just as accurately as is commonly done in the lower animals: these injections are absolutely safe and entirely trustworthy. As regards the X-rays, it has been found that they are "a decided help in the diagnosis of phthisis—a help which, as it becomes more generally used and its technique more thoroughly understood, will undoubtedly go hand-in-hand with physical examination at some future time."

The experience of the Brompton Hospital for consumption has often been quoted as evidence against the theory of **contagion.** Dr. James E. Pollock again summarises the evidence,[3] and considers that it negatives the idea of phthisis being an infective disease under such circumstances as being grouped in a hospital, breathing the same air, and living under the same conditions as others similarly affected. But, he adds, "later investigations have proved the fact that consumption is communicable, although not by personal contact, and that its existence depends on its transmission from one tuberculous individual (human or animal) to another. Such transmission is not aërial, but resembles more the process of inoculation."

The **sanatorium treatment** is yet in its infancy, although the open-air method was practised by Brehmer when Koch was still a school-boy. Three years' experience at Cromer has convinced Dr. Burton-Fanning of the practicability and utility of the open-air treatment in England, and he maintains that "an urgent necessity exists for the erection and maintenance in chosen parts of this kingdom of special institutions where the consump-

[1] *Practitioner*, 1898, lx. 590. [2] *Ibid.*, 602. [3] *Ibid.*, 609.

tive may be placed under the best conditions for recovery—where he may perpetually breathe the purest air, where everything that tends to the reinforcement of his resisting powers may be closely studied, and where, finally, he may be retained for a sufficiently long time."[1] Dr. Hermann M. Biggs, in his paper on the "Prevention and Restriction of Pulmonary Tuberculosis in the City of New York,"[2] shows what can be done, and his results show an almost regular and rapid decrease from year to year of the death-rate from all tubercular diseases; so much so, that he confidently anticipates the almost complete eradication of the disease as the result of continued efforts in this direction. Sanatorium treatment, equivalent to open-air treatment, is described by Dr. H. Weber,[3] who points out the difficulties attending the method, when carried on, under the ordinary circumstances of life, for a more or less prolonged period extending over several months, and not rarely over years. He advocates the establishment of sanatoria for the wealthier classes, for those without means of their own, and for those with slight means; also he considers it imperative to establish asylums for the incurable, where the end of their lives may be rendered as easy as possible, and he adds that those sanatoria ought not to depend entirely on benevolent contributions, inasmuch as the community has the duty to provide for the poor, and the trades' unions and workmen's insurances ought to contribute a share in their maintenance.[4] The treatment at Cromer by Dr. Burton-Fanning,[5] and that at Edinburgh by Dr. Philip,[6] are amongst the earliest attempts in this direction in England. That at Nordrach is described in *Diseases of the Lungs* by Dr. Kingston Fowler and Mr. R. J. Godlee.

**Treatment at high altitudes** has for many years been considered superior to any other, and Gardiner's researches on the immunity found at high altitudes were reviewed in our last number.[7] Dr. Theodore Williams sums up a large series of cases, and finds that no less a number than 83.4 per cent. show general improvement, 75.5 per cent. local improvement, and 42.5 per cent. absolute arrest or cure.[8] On the whole the evidence of the results of sanatorium treatment are far inferior to those given by Dr. Theodore Williams from high altitudes, and it seems doubtful whether anything else can produce those remarkable changes in the thorax and lungs, as well as those hæmatogenic changes, which high altitudes have been shown to do.

The **Mediterranean littoral** as a health-resort for phthisis is described by Dr. Michael G. Foster,[9] who makes the remark that as climatic stations for invalids the places mentioned are suited to some of the morbid phenomena attending pulmonary

[1] *Practitioner*, 1898, lx. 680. [2] *Ibid.*, 712. [3] *Ibid.*, 616.
[4] *Brit. M. J.*, 1898, i. 1164, 1229.
[5] *Lancet*, 1898, i. 630, 712, 854. [6] *Brit. M. J.*, 1898, ii. 217.
[7] *Bristol M.-Chir. J.*, 1898, xvi. 127.
[8] *Practitioner*, 1898, lx. 628. [9] *Ibid.*, 629.

phthisis, "but none of the southern climates can compare with that of the British Isles in their influence for developing the robust and maintaining them in the best possible condition."

The **desert climate** for lung tuberculosis is advocated by Dr. F. M. Sandwith,[1] and that of South Africa by Dr. Alfred P. Hillier,[2] who remarks that if "abundant sunshine, a dry, rarefied, and exhilarating air, and a pleasant, sub-tropical temperature be . . . conditions highly favourable to the phthisical subject, it must be freely admitted that there is no country in any of the five continents that offers these features in greater perfection than South Africa."

The **medicinal treatment** of tuberculosis, is summarised by Dr. Hector Mackenzie,[3] from which it appears that the creasote preparations in large doses still take the lead, although it cannot be shown that they have a true specific action.

Two other chapters on the **causation** of tuberculosis, and its prevention and restriction by legislative measures, bring this interesting and valuable collection of monographs to a close: they are all written by well-known authorities, and give us the latest information on a subject which demands imperatively the most patient and continued investigation by the best scientific and clinical observers.

The views of Dr. Loomis on **serum treatment** are summed up as follows: (1) Clinical facts have not borne out Koch's claim for his new tuberculin; (2) Hirschfelder's antitoxin does nothing; (3) Maragliano's serum gives no benefit and no harm; (4) Paquin's serum is certainly not inert; (5) Antistreptococcus serum gives no uniform improvement; (6) Neither the United States Government serum nor any one of the above-mentioned has any marked or immediate effect on the disease. Hirschfelder's oxytuberculin is well spoken of by Dr. Root,[4] of Monroe, Mich. He mentions three cases in which the results were very satisfactory. Not one of these cases could be classified as early, yet the manifest changes have been such as to point pretty certainly to the conclusion that in early cases this remedy will cure a large majority. Dr. Percy Kidd[5] remarks: "It is no exaggeration to say that in a further development and improvement of Koch's method lies our best hope of arriving at a successful treatment of tuberculous disease."

A **new method of treatment** is described by Dr. John B. Murphy[6] in his address before the American Medical Association in Denver, where he advocated the treatment of tuberculosis of the lung by the injection into the pleural cavity of large quantities of nitrogen gas. As much as one hundred and forty cubic inches were injected in one case: this was followed by slight dyspnœa, lasting, however, only a few minutes. Skia-

[1] *Practitioner*, 1898, lx. 640. [2] *Ibid.*, 649. [3] *Ibid.*, 681.
[4] *J. Am. M. Ass.*, 1898, xxx. 1502.
[5] Clifford Allbutt's *System of Medicine*, 1898, vol. v., p. 230.
[6] *J. Am. M. Ass.*, 1898, xxxi. 294, 341.

grams were taken at intervals of five weeks; and these showed that the gas was slowly removed. Inasmuch as 83 per cent. of cases of pleurisy were of tubercular origin, and that plastic pleurisy was curative of tuberculosis of lung, the method of treatment of tuberculosis of the lung by compression appeared to be a good one. The lung might be compressed for a long time, and would expand again; and there appeared to be little danger of any kind from simple pneumothorax. We must await further evidence on this novel and enterprising proceeding.

Dr. Henry P. Loomis,[1] New York, writes on certain points of interest which are of help in estimating **the future of a case** of phthisis:—1. *Gastric symptoms.* "Climate avails little when the patient is daily losing ground because of unassimilated food. Medication is worse than useless, because of an enfeebled digestion which is constantly disturbed." 2. *Rapid heart-action.* "In the advanced stages of the disease we expect to find a rapid and often feeble heart, but when there is only slight involvement of the lung, and the heart action is always above normal even at rest, and when on the slightest exertion it goes up to 110 or 120 beats, the outlook is always unfavorable." 3. *Age.* "My experience has been that the phthisis of advanced age is often latent in its beginnings, slow in its advance, tending to the destruction of limited portions of the lungs, and is found with few secondary derangements accompanying it." 4. *Fibroid diathesis* is an element of favorable prognosis. 5. *Hæmoptysis* early in the disease does not affect the prognosis one way or the other. 6. *Heredity* has very little to do with the question of a person's recovery, all other things being equal. 7. *Habits.* Alcoholic subjects do badly when they contract phthisis; the resisting power of their tissues seems to be at a minimum; their course downward is generally rapid. 8. *Loss of Flesh.* Unless a person gains in weight he is not improving: this is one of the best tests that we have. 9. *Character.* The tediousness of recovery from tuberculosis, at the best, requires all the fortitude and perseverance of which a strong character is capable. 10. *As regards the lung condition*, it seems that those cases in which tuberculosis develops secondary to a subacute pleurisy or is co-existent with marked changes in the pleura, uniformly do well when placed under suitable climatic conditions.

R. SHINGLETON SMITH.

